# Metagenomic Sequencing in the ICU for Precision Diagnosis of Critical Infectious Illnesses

**DOI:** 10.1186/s13054-023-04365-1

**Published:** 2023-03-21

**Authors:** Lucile P. A. Neyton, Charles R. Langelier, Carolyn S. Calfee

**Affiliations:** 1 Division of Pulmonary, Critical Care, Allergy and Sleep Medicine, Department of Medicine, University of California San Francisco, San Francisco, CA USA; 2 Division of Infectious Diseases, Department of Medicine, University of California San Francisco, San Francisco, CA USA; 3 Chan Zuckerberg Biohub, San Francisco, CA USA

## Abstract

This article is one of ten reviews selected from the Annual Update in Intensive Care and Emergency Medicine 2023. Other selected articles can be found online at https://www.biomedcentral.com/collections/annualupdate2023. Further information about the Annual Update in Intensive Care and Emergency Medicine is available from https://link.springer.com/bookseries/8901.

## Introduction

Infectious diseases, in particular respiratory and bloodstream infections, are a leading cause of intensive care unit (ICU) admission and death worldwide [1]. Identifying the underlying pathogens responsible for infectious critical illness remains a major challenge and delays timely and effective treatment. Indeed, pathogens remain undetected in up to 60% of cases of pneumonia [2] and over 30% of cases of sepsis [3, 4]. Appropriate antibiotic therapy is essential for effective management of critical infectious diseases; however, in most cases, treatment is empiric because existing microbiologic diagnostics are unable to identify an etiologic pathogen. This approach also contributes to antimicrobial resistance, opportunistic pathogens such as *Clostridium difficile*, and leads to other avoidable adverse drug effects [5, 6]. Rates of antimicrobial-resistant infections have markedly increased during the coronavirus disease 2019 (COVID-19) pandemic due in part to the overuse of broad spectrum antibiotics from clinicians suspecting secondary bacterial infections but lacking diagnostics to confidently determine their existence [7, 8]. Thus, improvement in diagnostics for pathogens causing infectious illness in critically ill patients remains a major unmet need.

Metagenomics, the study of nucleotide sequences from all organisms in biological samples, offers an unprecedented opportunity to rapidly identify and characterize infectious disease-causing pathogens, such as bacteria, viruses, and fungi, in a single test without a need for culture. The term metagenomics traditionally refers to DNA sequencing, whereas metatranscriptomics refers to RNA sequencing. However, the term metagenomics is commonly used to refer to DNA *and* RNA sequencing, both of which can be used for pathogen detection, with important differences and associated considerations. In this review, we will use the term metagenomics to refer to both DNA and RNA sequencing.

This chapter begins with providing an overview of the current metagenomic approaches used to identify pathogens. Next, we will describe examples of metagenomics applications and examine how the technique might be employed more widely to study and treat infectious diseases in the ICU.

## Current Standards in Pathogen Detection

Historically, the gold standard for identification of bacterial and fungal pathogens has been culture [9]. Despite simplicity and low cost, the turnaround time for culture-based methods can extend up to several days or even weeks [10], leading to delayed diagnoses, inappropriate antimicrobial use, and in some cases excess disease transmission in the hospital due to missed infections [11]. While standard blood and respiratory cultures are relatively inexpensive compared to many medical diagnostic tests, in some countries, such as the USA, the cost of labor and routine use of mass spectrometry for taxonomic identification have led to per-patient costs of several hundred US dollars. Viral pathogens and some bacterial pathogens, such as *Mycoplasma pneumoniae* or *Legionella pneumophila*, may be difficult to detect with traditional culture-based methods [12]. Because empirical antibiotic treatment is typically administered as early as possible in patients presenting with infection-related symptoms, the use of culture-based identification might also lead to false negative results as antibiotics can sterilize microbial cultures.

Immunological methods, such as serology, can also be used to determine the presence of antibodies directed at the pathogen of interest. The major drawback of using immunological assays for the detection of pathogens is that antibody production requires several days to weeks following exposure to a pathogen, leading to false negative tests during the period of acute illness [13]. Antigen tests directly detect pathogen proteins and do have utility during acute illness; however, they are only available for a limited number of organisms and in many cases have limited sensitivity and/or specificity [13].

Viral detection, and increasingly *Mycobacterium tuberculosis* detection, is carried out using polymerase chain reaction (PCR) assays. Many pathogen genomes have been sequenced and are publicly available, which allows the design of species-specific probes that can be used to find and amplify microorganism-specific nucleic acid sequences, thus allowing the targeted detection of a set of pre-defined micro-organisms, often within just a few hours [14]. However, despite the availability of many Food and Drug Administration (FDA)-approved microbial tests [15] allowing the identification of a range of different pathogens (bacteria, viruses, fungi, and parasites), only a handful of PCR-based assays are clinically accepted and available in routine practice, and less common organisms, novel emerging pathogens, or pathogen variants may be undetectable using such approaches.

All these methods are targeted, meaning that they focus on a pre-selected set of organisms. In many cases, only common pathogens are sought, thus limiting the chances of identifying less common pathogens of interest.

## Principles of Metagenomics for Infectious Disease Diagnosis

The potential of metagenomics to improve infectious disease diagnosis in the ICU, where time to effective treatment is paramount [11], is significant. Metagenomics allows the unbiased detection, quantification, and characterization of genetic material from any organism within biological samples in a relatively short timeframe (Table 1)

**Table 1:** Characteristics of commonly used pathogen identification strategies

Identification method	Principle	Cost	Microbial detection	Additional considerations	Turnaround
Culture	Growth and isolation of species present in a sample	Low-moderate	Some species are difficult to culture or cannot be cultured (e.g., atypical organisms, viral, fungal pathogens)	• Medium-dependent • Prior use of antimicrobial agents will affect sensitivity	Days to weeks
Immunological methods	Detection via antibodies	Low-moderate	Determined by the choice of antibody/ antigen	• Antibody testing may not be useful during acute disease	Minutes to days
	Detection via antigens			• Limited by sensitivity/ specificity	
PCR	Targeted amplification of specific pathogens	Moderate	Limited by PCR primer panel	• Detects only a few pre-selected microbes • Some species might be preferentially amplified	Minutes to days
Metagenomics	Nucleotide sequences capture and amplification	High	Unbiased	• Host background will be dominant • Contamination will greatly affect utility	Hours to days

*PCR* polymerase chain reaction

**Fig. 1:**
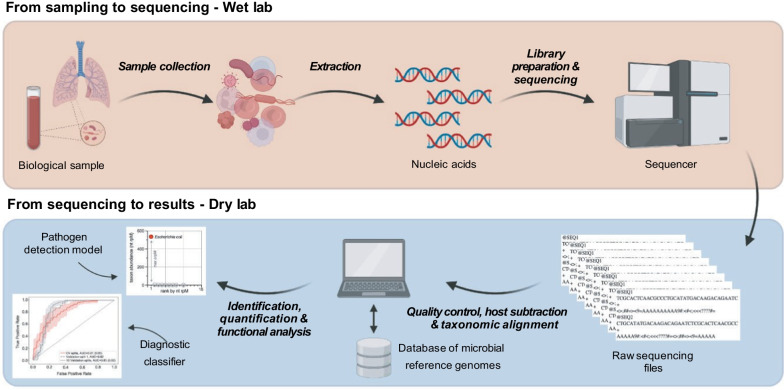
Simplified overview of a metagenomics workflow, which is broken down into two main steps. Sample collection, nucleic acid extraction, library preparation, and sequencing are depicted in the orange panel. Once reads are sequenced, data are fed into a bioinformatics pipeline (blue panel) for quality control, host subtraction, and taxonomic alignment, followed by identification and quantification of microbial species, and functional analysis. Two possible analyses are depicted and consist of pathogen detection and disease classification (figures adapted from Kalantar et al. [16]). Created with BioRender.com

The general metagenomics workflow (Fig. 1) begins with nucleic acid extraction (DNA and/or RNA) from the biological sample of interest. This step is followed by library preparation, during which nucleic acid is fragmented, and short adapter sequences are ligated onto the ends of the fragments to permit PCR amplification and binding to the sequencer flow cell. Samples are typically barcoded to enable multiplexing. Long-read (e.g., Oxford nanopore, Oxford, UK) and short-read (e.g., Illumina, San Diego, CA, USA) sequencing platforms can be used clinically, with turnaround times ranging from 6 h to several days depending on instrumentation, degree of sample multiplexing, and infrastructure [17].

Prior to analysis, raw sequencing reads must be demultiplexed based on barcodes, filtered for quality and complexity, and trimmed to remove adapters and barcodes. The resulting sequencing data contains both host and non-host (i.e., microbial) components, which vary in proportions depending on type of biological specimen, though host data often represent the vast majority. The host reads can either be discarded from further analysis or, in the case of RNA sequencing, analyzed to assess host gene expression. To identify microbial taxa present in the sample, non-host sequences are aligned to reference databases, such as the NCBI nucleotide database, containing reference pathogen genomes. In cases of novel pathogens, reference database alignment will be imperfect, but generally capable of providing insight regarding the most similarly related microbes. Alternatively, to detect species and strains that might not be present in the reference database, a *de novo* assembly and annotation approach can be taken.

Additionally, quantification can be performed to estimate the relative abundance of different taxonomic groups, and functional analysis can be carried out (Fig. 1). Functional analysis can involve the identification of antimicrobial resistance and/or virulence factor genes, using for example publicly available databases.

## DNA Sequencing vs. RNA Sequencing

DNA sequencing is considered the usual method of choice for the detection of pathogens in a range of different sample types [18] because it targets all DNA present in a sample and will capture non-actively transcribed or non-functional genes as well, providing additional taxonomic and functional information. However, DNA sequencing will not allow detection of RNA viruses, as only DNA will be amplified during the sequencing process. Conversely, metatranscriptomics can be used to detect RNA as well as replicating DNA viruses and might thus allow a broader detection of pathogens. For the detection of bacterial species when performing RNA sequencing, even though more bacterial sequences will be detected, differences in bacterial transcript abundances might lead to fewer species being detected as a species might be contributing more transcripts than others [19]. To add more complexity, organisms detected via DNA sequencing might not reflect active infection, but may instead represent nonviable organisms and/or environmental deposition [20]. For researchers interested in the interplay between pathogens and the host response, RNA sequencing enables simultaneous sequencing of pathogens and host gene expression from a single sample to provide a comprehensive snapshot of interactions [21].

While each sequencing approach provides complementary and valuable information, conducting both DNA and RNA sequencing is often prohibitively expensive and/or time-consuming. In essence, the decision to sequence one or the other should be carefully considered in the early phases of the project and should be based on the questions and samples of interest.

## Proof of Concept and Clinical Trial Data for Metagenomic Diagnostics

Metagenomic strategies have been successfully used for the diagnosis of infecions in critically ill patients using a variety of sample types, such as cerebrospinal fluid (CSF) to identify meningitis and/or encephalitis [22–24], circulating blood to identify sepsis [18, 24], and respiratory samples (tracheal aspirate [25] and bronchoalveolar lavage [BAL] [23, 24]) to diagnose lower respiratory tract infections, among others.

In one of the initial demonstrations of the clinical utility of this approach, metagenomics for diagnosis of central nervous system infections in CSF samples was investigated in 204 severely ill hospitalized patients [22]; 58 infections were identified, 13 of which had not been identified via clinical testing but were solely diagnosed using metagenomics testing. In seven of these cases, the results of metagenomics testing led to clinically impactful changes in antibiotic treatment (i.e., extension, narrowing, or adjusting of spectrum) and enabled timely resolution of the infection. Notably, metagenomic testing also had a significant false negative rate, with 26/58 (45%) clinically confirmed infections not detected by metagenomic sequencing. Gu and colleagues [23] reported the results of metagenomic sequencing in 182 samples from 160 patients with acute illness, with comparison to culture and PCR testing as the gold standard for infection diagnosis. Body fluid samples included abscess aspirate, synovial fluid, pleural fluid, ascites, CSF, BAL, and others. In this dataset, the sensitivity of metagenomic sequencing for bacterial infection ranged from 75% to 79% (depending on the sequencing method), with specificity of 81–91%, with even higher sensitivity and specificity for fungal species. With the important exception of plasma, metagenomic sequencing appeared to perform well across body fluid sample types studied.

The diagnostic utility of metagenomics has also been studied in sepsis. In one cohort of 350 patients [18] a 94% concordance between blood culture and plasma-based metagenomics testing was reported. Metagenomics also permitted the identification of disease-causing organisms in more cases than culture (169 vs. 132, respectively). In another study of 193 patients with sepsis, a higher rate of pathogen detection was reported using metagenomics (85%) when compared to culture (31%) [24]. In that study, concordance for metagenomics testing and culture was 30%, and 55% of microbial species were detected solely with metagenomics. These results were consistent across several samples, including CSF, circulating blood, and BAL. Of note, in this study, metagenomics showed high detection rates for bacteria and viruses, but lower rates than culture when considering fungal species such as *Candida*.

Metagenomics has also been evaluated for the diagnosis of lower respiratory tract infections in the ICU using BAL samples. In one study of 22 hematopoietic stem cell transplant patients [25], identification of a putative pathogen was reported in 12 patients; 6 had not been detected using routine clinical diagnostic tests. Another larger study of lower respiratory tract infection in 92 patients with acute respiratory failure found that metagenomic analyses of tracheal aspirate could identify pathogens with 96% accuracy compared to culture, and also identify putative missed pathogens in over 60% of cases with clinically suspected lower respiratory tract infection but negative standard of care microbiologic testing [26]. More recently, a similar study focusing on children with lower respiratory tract infection investigated the use of metagenomics for diagnosis and pathogen identification in 397 individuals [27]. In that analysis, the disease-causing organism was identified in 92% of lower respiratory tract infection cases, and the integration of clinical testing and metagenomics enabled a diagnosis in 90% of cases vs. 67% for routinely ordered testing.

An overview of these studies and selected additional exemplary clinical investigations of metagenomic studies is presented in Table 2.

**Table 2:** Case examples using metagenomics for the diagnosis of infectious disease and identification of disease-causing organisms

Disease of interest	Samples	Studies [Ref]
CNS infection	CSF	Wilson et al. [22] Gu et al. [23]
Sepsis	Plasma	Blauwkamp et al. [18] Ren et al. [24] Kalantar et al. [16]
Respiratory infection	BAL Pleural fluid Tracheal aspirate	Gu et al. [23] Langelier et al. [25] Langelier et al. [26] Tsitsiklis et al. [27]
Abscess	Abscess fluid	Gu et al. [23]
Peritonitis	Peritoneal fluid	Gu et al. [23]
Urinary tract infection	Urine	Gu et al. [23]
Septic arthritis	Joint fluid	Gu et al. [23]

*CNS* central nervous system, *CSF* cerebrospinal fluid, *BAL* bronchoalveolar lavage

## Metagenomics for Prediction of Pathogen Antimicrobial Resistance

Antimicrobial resistance is one of the most urgent threats to human health and a major challenge for managing infections in the ICU [28, 29]. Historically, detection of antimicrobial resistant pathogens has necessitated phenotypic susceptibility testing of clinician-ordered bacterial cultures. Direct detection of antimicrobial resistance gene products through metagenomics offers an opportunity to overcome the limitations of culture by directly detecting the pathogen genes conferring antimicrobial resistance. Databases such as the Comprehensive Antibiotic Resistance Gene Database (CARD) [30] can map reads to known antimicrobial resistance genes from a diverse set of organisms [31]. Further, some bioinformatics pipelines, such as the ID-seq pipeline [32], enable integrated taxonomic and antimicrobial resistance gene identification. Metagenomics has been employed in hospital settings to study the distribution of resistant organisms [33–35], and a recent proof of concept study demonstrated utility for antimicrobial resistance prediction in critically ill patients with pneumonia [29]. Advances in machine learning algorithms may ultimately enable genotype to phenotype prediction for a broad range of organisms, although limitations in genome coverage of low abundance resistance genes in metagenomic datasets are currently an important barrier to overcome [36]. Metagenomics holds promise for expanding the functionality of existing public health surveillance systems by enabling surveillance for known and emerging antimicrobial resistant pathogens in the hospital, community, and environment [31].

## Assessing the Host Response to Enhance Metagenomic Pathogen Detection

In most metagenomic approaches, only host or only microbial data is generated and analyzed, permitting *either* the detection of microbial species *or* the profiling of the host response. However, capturing *both* components with RNA sequencing, which can enable pathogen detection *and* profiling of the host response, can provide a more complete picture of the complex interplay between pathogens and host. In the context of infection, it can be challenging to distinguish commensals from disease causing organisms; however, combining pathogen identification data with host response profiling can help with this distinction.

Two recent studies have reported approaches integrating microbe and host response to improve diagnosis and understand infectious diseases in lower respiratory tract infections and sepsis, respectively [16, 25]. In the study of 92 respiratory failure patients described earlier [25], a combined microbe and host signature was employed to distinguish lower respiratory tract infections from non-infectious etiologies of respiratory failure in tracheal aspirate samples. This approach also identified pathogens and recognizing pathogens from commensal organisms, because of the complimentary of the datasets, was further enhanced by integrating the host-derived data. In integrating host and microbe data, cases of infection were diagnosed with high accuracy (96%). Another recent study took a similar approach to sepsis diagnostics, integrating host and microbe data from blood metagenomic and metatranscriptomic sequencing of 221 critically ill patients for a diagnosis of sepsis and identification of pathogens in blood samples [16]. Notably, the integrated metagenomic model identified 99% of sepsis cases with positive microbiology, predicted sepsis in 74% of the suspected sepsis cases with negative conventional microbiology, and was consistent with a diagnosis of sepsis in 89% of unclear sepsis cases. Furthermore, patients without sepsis were correctly predicted as non-sepsis with a specificity of 78%, highlighting the model’s potential utility as a rule-out diagnostic test. This proof-of-concept study highlighted the potential of integrating host and microbe data to diagnose sepsis and identify relevant pathogens, especially for cases without positive microbiology or more complex cases.

## Metagenomics: Potential Hurdles and Important Considerations

In addition to choosing the sample to perform the sequencing on and the type of sequencing (DNA- vs. RNA-sequencing), there are some limitations, challenges, and important questions to consider when considering a metagenomics-based approach for the detection of pathogens in the ICU. First, metagenomics-based approaches permit the detection of not only relevant pathogens, but also all low abundance commensal and environmental contaminating organisms that may be present in a sample. Identifying commensal organisms is especially relevant in the context of non-sterile-site samples (e.g., lung and gut) that contain complex microbial backgrounds, as opposed to typically sterile samples such as CSF. Recent advances in algorithms to distinguish pathogenic microbes from commensal or contaminating organisms have been an important step to interpreting the significance of the hundreds of microbial alignments that result from analysis. One algorithm, for example, is designed to identify disproportionately abundant microbes within samples and only report those with established pathogenicity [25]. For all samples, to ensure the taxonomic alignments detected are relevant and not due to environmental contaminants, both negative (water or synthetic matrix) and positive controls must be included and processed in the same way as test samples [37].

Second, the proportion of host-derived sequences in metagenomic data can be quite high, ranging from 10% (gut) to over 95% (respiratory) of total sequences depending on the anatomical site of sampling [38]. If the goal of sequencing is to detect pathogens alone, then increasing coverage by generating more sequencing reads or using targeted enrichment methods [39] should be considered, though these approaches will increase cost and complexity. A larger proportion of host nucleotide sequences will lead to decreased sensitivity for microbial detection due to lower coverage of non-host sequences [40].

Third, metagenomics remains a costly diagnostic approach that has not yet been incorporated into standard of care in most clinical settings. Despite an increased cost with respect to culture- or PCR-based methods, clinically practical metagenomics assays have comparable costs (~2000 US dollars) to a computed tomography (CT) scan with contrast. While this cost is still a major barrier in many settings, particularly in low- and middle-income countries, sequencing costs continue to decrease each year as technology improves [41]. Historically, intensive computational requirements have also been a barrier to the broader clinical use of metagenomics assays; however, the availability of free, cloud-based bioinformatics pipelines [32] has democratized the bioinformatics steps needed to go from sequence to pathogen identification.

## Conclusion

Despite promising results, metagenomics remains underutilized in the ICU. Several factors still limit its inclusion in routine critical care, including the lack of definitive clinical trials testing its utility, few laboratories with the infrastructure needed to afford rapid turnaround, cost in low resource settings, and the fact that few metagenomics assays have undergone the clinical validation needed to permit use in patient care. These barriers will need to be overcome before wide adoption of metagenomics into clinical practice. However, an increasing number of studies are demonstrating the potential utility of metagenomics in a range of settings relevant to critically ill patients. Moving forward, a gradual inclusion of metagenomics into current clinical diagnosis pipelines, starting from a complementary inclusion along with currently used tests in severely ill patients, may demonstrate the full potential of this technology in the ICU.

## Data Availability

Not applicable.
